# Prevalence of Anxiety and Depression among Psychiatric Healthcare Workers during the COVID-19 Pandemic: A Malaysian Perspective

**DOI:** 10.3390/healthcare10030532

**Published:** 2022-03-14

**Authors:** Mehul Kumar Narendra Kumar, Benedict Francis, Aili Hanim Hashim, Nor Zuraida Zainal, Rusdi Abdul Rashid, Chong Guan Ng, Mahmoud Danaee, Nurulwafa Hussain, Ahmad Hatim Sulaiman

**Affiliations:** 1 Department of Psychological Medicine, University Malaya Medical Centre, Kuala Lumpur 59100, Malaysia; mehulkumar32@siswa.um.edu.my (M.K.N.K.); benfrancis@um.edu.my (B.F.); ailihas@um.edu.my (A.H.H.); norzuraida@um.edu.my (N.Z.Z.); rusdi@um.edu.my (R.A.R.); chong_guan@um.edu.my (C.G.N.); 2 Faculty of Medicine, University Malaya, Kuala Lumpur 50603, Malaysia; 3 Department of Social and Preventive Medicine, Faculty of Medicine, University Malaya, Kuala Lumpur 50603, Malaysia; mdanaee@um.edu.my; 4 Department of Psychiatry and Mental Health, Hospital Melaka, Jalan Mufti Haji Khalil, Melaka 75400, Malaysia; nurulwafahussain@gmail.com

**Keywords:** COVID-19, anxiety, depression, coping, mental health, psychiatry, non-frontline, healthcare workers, Malaysia

## Abstract

The psychological distress reported among frontline healthcare workers (HCWs) is concerning. Little is known about the mental health of non-frontline, psychiatric HCWs, who play a central role in handling the mental health crisis during the COVID-19 pandemic. This study aimed to examine the prevalence of anxiety and depression among psychiatric HCWs and evaluate its association with socio-demographic, socio-economic, work-related factors and coping strategies. The authors proposed a cross-sectional study design using the Hospital Anxiety and Depressive Scale (HADS) and Brief-COPE scale. This study found that the prevalence of anxiety and depression were 22.0% and 16.8%, respectively. A multivariate analysis revealed that married psychiatric HCWs had a lower level of anxiety with OR = 0.31 (95% CI: 0.11–0.83). Psychiatric HCWs who were experiencing financial hardships, were unvaccinated and those who had a shorter duration of service in the psychiatric department had a higher level of depressive symptoms with OR = 0.31 (CI: 1.19–11.27), 3.21 (CI: 0.97–10.52), and 1.01 (CI: 1.00–1.02), respectively. For every increase of one unit of avoidant coping score among respondents, the odds of having anxiety and depression increased by 1.25 times (CI: 1.15–1.37) and 1.20 times (CI: 1.09–1.32), respectively, whereas for every increase of one unit of religious coping score among respondents, the odds of having anxiety reduced by 1.42 times (CI: 1.10–1.84). The authors highlight that psychosocial measures addressing the relatively high levels of anxiety and depression among psychiatric HCWs should be a key priority to ensure the sustainment of mental health services in the face of this prolonged pandemic.

## 1. Introduction

It has been almost two years since coronavirus disease 2019 (COVID-19) emerged in Wuhan City, Hubei Province, China on 12 December 2019. Malaysia saw its first COVID-19 case on 25 January 2020. The World Health Organization (WHO) officially declared COVID-19 a global pandemic on 11 March 2020 [1]. Despite almost two years since the emergence of COVID-19, the number of new global cases is still at concerning levels. As of 1 October 2021, COVID-19 has caused more than 234 million cases and 4.7 million deaths globally, with more than 2.2 million cases and 26 thousand deaths attributed to COVID-19 within Malaysia alone [2,3].

Similar to many other countries, to mitigate the local spike of COVID-19 infection, the Malaysian government announced the first movement control order (MCO), starting on 18 March 2020. The MCO incorporated several important measures, namely, the implementation of border control, control of public movement, prohibition of public gathering and enforcing of physical distancing. At the time of writing, Malaysia is still experiencing the third phase of the MCO. Undoubtedly, the COVID-19 pandemic and the prolonged imposed movement control order in Malaysia has significant economic, social, and mental health consequences [4]. 

Studies conducted locally and internationally during the COVID-19 pandemic began to show alarming levels of psychological distress in the community [5,6,7,8]. A report by the Center for Disease Control [6] showed that 40.9% of the 5470 studied adult respondents reported at least one adverse mental or behavioural health condition. Azuddin et al. [7] conducted a study among Malaysians in February 2021 and found 56% and 58% of the respondents experienced anxiety and depressive symptoms, respectively. Forty-two per cent of the respondents in this study also felt that their mental state had worsened compared to the same time the previous year. 

Healthcare workers (HCWs) are also at risk of experiencing psychological distress as they continue to face the enormity and uncertainty of the pandemic, aggravated by limited resources [9,10]. Sahebi et al. [11] documented, in a review of seven meta-analysis studies consisting of 108 articles and 433,800 healthcare workers, that the prevalence of anxiety and depression among frontline HCWs during the COVID-19 pandemic was 24.94% and 24.83%, respectively. 

Studies conducted primarily among frontline HCWs during the COVID-19 pandemic revealed multiple factors associated with anxiety and depression. Alnazly et al. [12] documented that female, elderly (>40 years old), and married HCWs living with family had higher levels of anxiety and depression. In a study conducted in China evaluating the mental health of medical HCWs during the pandemic, Zhang et al. [13] found that having an organic disease was an independent factor for insomnia, anxiety, depression, somatisation, and obsessive–compulsive symptoms. Meanwhile, a study in Turkey by Ilhan and Kupeli [14], during the COVID-19 pandemic, found that HCWs with financial difficulties were at the highest risk of developing anxiety, depression, and secondary traumatic stress. 

Studies evaluating work-related factors revealed that HCWs with longer working experience (>20 years) [12], and those who provided direct care to COVID-19 positive patients had higher levels of anxiety and depression [12,15,16]. An increased work burden due to the pandemic was also associated with increased psychological distress [10,17,18]. HCWs who were unvaccinated [19], working long hours (>15 hours/day) [20], and those investigated as close contacts, irrespective of the results of COVID-19 tests [21], showed higher levels of psychological distress. Occupational differences, with nurses exhibiting higher rates of anxiety and depression compared to other HCWs, were documented in two systematic reviews and meta-analysis by Pappa et al. [10] and Marvaldi et al. [22]. 

Coping is recognised both for its significant impact on mental and physical health outcomes and for its intervention potential. Taylor and Stanton [23] described coping as an action-oriented intrapsychic effort to manage the demands created by stressful events. The coping approach involves behavioural, thought, or affective actions oriented towards a stressor. Conversely, the avoidant approach is defined as any action or confronting emotional response that is oriented away from a stressor [24]. Religious coping strategies rely on a secure relationship with God/the divine [25]. Some studies demonstrated that avoidant coping is positively correlated with anxiety and depression, while approach coping and positive religious coping strategies are correlated inversely with anxiety and depression [26,27,28,29]. Abel [30] described the humour coping strategy as producing a cognitive–affective shift or restructuring tensed situations that are less threatening, with a concomitant reduction in physiological arousal. Thus, coping strategies for HCWs could either buffer or intensify their psychological distress during the pandemic [26,27,28,29].

The increasing demand for mental health services due to the COVID-19 pandemic comes at a time following a steep increase in mental health difficulties in the Malaysian population over the last few decades. The prevalence of poor mental health among Malaysian adults has steadily increased from 10.7% in 1996 to 11.2% in 2006 and 29.2% in 2015 [31]. Despite the enormous psychological burden in the community, Malaysia’s mental health workforce is limited. In 2018, there are only 410 registered psychiatrists in Malaysia, resulting in a psychiatrist-to-population ratio of 1.27 per 100,000 people [32]. The ratio is a far cry from the WHO recommendation of one psychiatrist for every 10,000 people [33]. Correspondingly, Suarn et al. [34] reported that psychiatric nurses’ and psychologists’ ratios were also low: 3.31 and 0.29 per 100,000 people.

Psychiatric HCWs play a central role in meeting the increasing demand for mental health services. In most settings in Malaysia, psychiatric HCWs are primarily responsible for providing psychological interventions to other HCWs and the population at large [32]. Considering the psychological impact of the pandemic and the increasing demand for mental health services, psychiatric HCWs may also be predisposed to mental health problems themselves. However, little is known about the mental health of psychiatric HCWs. This is likely due to the assumption that psychiatric HCWs are mentally robust; they should be adequately equipped with the skills and knowledge to handle the psychological effects of the pandemic [32]. This assumption may not be correct. A high level of undetected anxiety and depression among psychiatric HCWs would significantly impact their ability to provide care for the increasing number of patients facing psychological distress because of the pandemic.

To our knowledge, there are no studies to date assessing the psychological impact of the COVID-19 pandemic among the psychiatric HCWs in Malaysia. The study aimed to evaluate the prevalence of anxiety and depression among psychiatric HCWs. The study also aimed to determine the socio-demographic, socio-economic, and work-related factors, as well as coping strategies associated with anxiety and depression among psychiatric HCWs. The findings of the study may assist in identifying the at-risk psychiatric HCWs who will benefit most from the psychosocial interventions and help improve mental health service planning in general. 

## 2. Materials and Methods

The cross-sectional study was conducted between 1 May 2021 and 31 August 2021. The study participants were psychiatric HCWs working at the Department of Psychological Medicine, University Malaya Medical Centre (UMMC). UMMC is a teaching hospital located in Kuala Lumpur, the epicentre of the COVID-19 pandemic in Malaysia. Since the pandemic started, the Malaysian Government designated UMMC as a hybrid hospital to manage COVID-19 cases in Malaysia. UMMC has 1600 beds, covering a comprehensive range of medical specialities.

The study utilised a universal sampling method. All psychiatric HCWs (n = 196) working at the Department of Psychological Medicine UMMC were invited to participate in the survey via instant text messages in the department WhatsApp groups during the study period. Given the pandemic, and to minimise the risk of transmission, the researchers never met the participants personally, and the study was hosted online using Google Forms. Participants who voluntarily consented to participate in the study, met the inclusion criteria, and did not meet the exclusion criteria listed online were directed to the self-administered standardised questionnaires with instructions hosted on Google Forms.

Individuals who completed the online survey (n = 177) were screened once again by the researchers based on inclusion and exclusion criteria. Four individuals were excluded as they had worked for less than two months in the Department of Psychological Medicine UMMC before participating in the study. We set a minimum of two months working duration at the department to give adequate time for the psychiatric HCWs to assimilate and be adequately adapted to the work-related factors in the department.

**Figure 1:**
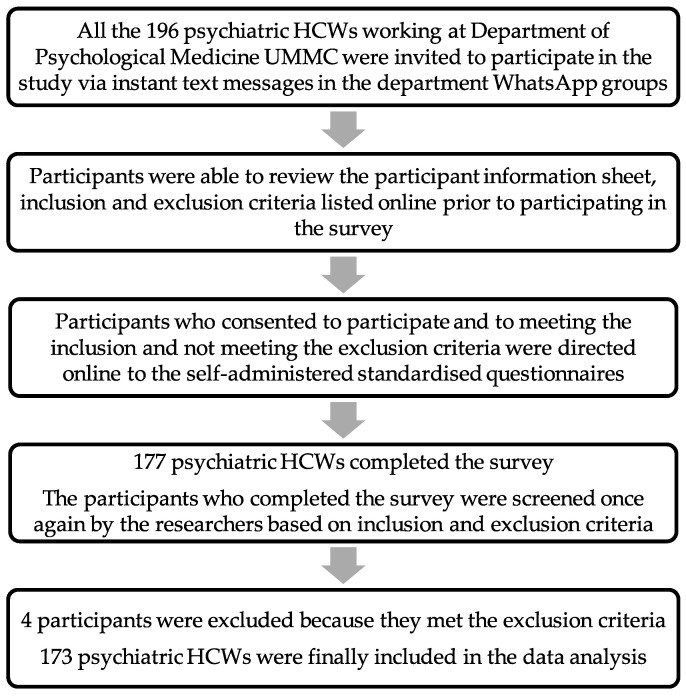
Flow chart describing the methodology of the study.

Therefore, 173 psychiatric HCWs who met the inclusion criteria and did not fulfil the exclusion criteria were included in the analysis of this study. Figure 1 displays the flow chart describing the methodology of the study. 

### 2.1. Inclusion Criteria

- Psychiatric HCWs who were working at the Department of Psychological Medicine UMMC during the study period from 1 May 2021 till 31 August 2021;
- Available to be contacted via instant text message;
- Able to understand English or Malay language;
- Able to give consent.

### 2.2. Exclusion Criteria

- Under the age of 18 years old;
- Psychiatric HCWs who had worked for less than two months at the Department of Psychological Medicine UMMC before participating in the study;
- Medical and nursing students who were completing a placement at the Department of Psychological Medicine UMMC during the COVID-19 pandemic;
- Declined to participate in the study.

### 2.3. Measurement Tools

The potential covariates for the study were selected based on significant findings from the literature evaluating anxiety and depression among frontline HCWs [10,12,13,14,15,16,17,18,19,20,21,22,26,27,28,29,30]. A questionnaire was designed for the study to collect the participants’ socio-demographic, socio-economic, and work-related data. Participants coping strategies were assessed using the Brief-COPE Scale. The outcome variables of depression and anxiety were measured using the Hospital Anxiety and Depression Scale (HADS). Participants could answer the questionnaire either in English or Malay, the Malaysian national language.

#### 2.3.1. Hospital Anxiety and Depressive Scale (HADS) English and Malay Version

The HADS is a self-reported questionnaire designed to screen for anxiety and depression among the respondents [35]. The scale consists of 14 items in a mixed arrangement. Seven items address anxiety symptoms (HADS-A), and the other seven are related to depressive symptoms (HADS-D). Each item has a score of 0 to 3. The anxiety and depressive domain scores can range from 0 to 21. The conventional cut-off score of the scale is 11. To avoid missing out a significant fraction of the Malaysian population with anxiety and depressive symptoms, a lower cut-off score of 8 was used in this study, as it was shown to have a sensitivity of 93.2% and specificity of 90.8% based on a locally conducted study by Yahya and Othman [36]. Respondents who scored eight and above were further categorised into mild anxiety or depression (score 8–10), moderate anxiety or depression (score 11–14), and severe anxiety or depression (score 15 and more) [36]. The translated Malay version of this scale has a Cronbach’s alpha of 0.87 [37].

#### 2.3.2. Brief-COPE English and Malay Version

The Brief-COPE Scale is a 28-item self-rated questionnaire to assess coping strategies among respondents when faced with stressful situations [38]. It has 28 items rated by a four-point Likert scale. These 28 items are further categorised into 14 item subscales, with two items per subscale representing different coping strategies. The minimum and maximum Brief-COPE total scores for each item subscale are 2 and 8, respectively. The total score for each subscale was used for further analysis. These 14-item subscales can be further analysed based on different factor models [39]. For the analysis, the study used the 4-factor model, which is supported by a fundamental coping theory by Carver [38]. The 4-factor model categories are approach coping, avoidant coping, religion, and humour [38,40]. The approach coping category encompasses the subscale items of acceptance, planning, positive reframing, active coping, information support, and emotional support. In contrast, the avoidant coping category contains the subscale items of self-distraction, venting, denial, behavioural disengagement, self-blame, and substance use. The humour and religion subscale items are stand-alone categories and are analysed independently. The Brief-COPE has an internal consistency (Cronbach alpha) of 0.70 overall and 0.44–0.89 for the 14 subdomains. Both the English and Malay versions were validated in Malaysia. The translated Malay version of this scale has a Cronbach alpha of 0.83 [26]. 

### 2.4. Statistical Analyses

Statistical Package for Social Sciences (SPSS) version 27 was used for data analysis. A descriptive statistic was carried out to summarise the independent and dependent variables. Normality testing was conducted through a combination of both statistical (continuous variables, with values of skewness and kurtosis less than 2.00 considered to be normally distributed) and graphical modalities (boxplots). The bivariate analysis examined the association between the covariates (socio-demographic, socio-economic, work-related factors, coping strategies) and the dependent variable (anxiety and depression). All covariates with a *p*-value of less than 0.25 from the bivariate analysis were subjected to multivariable logistic regression analysis, using the forward-stepwise regression method. Variables with a *p*-value < 0.05 are considered to be significantly associated with anxiety and depression. 

### 2.5. Ethical Considerations

All participants participated in the study voluntarily and had the opportunity to review the participant information sheet online before participating. No identifiable details such as name, email, and contact number were collected from the participants to ensure anonymity. The respondents who believed they were psychologically distressed were encouraged to contact the principal investigator or visit the nearest health care facility for further evaluation and treatment. This study was approved by the Medical Research Ethics Committee of UMMC (MREC 202123-9795).

## 3. Results

A total of 173 out of the 196 psychiatric HCWs working at the Department of Psychological Medicine UMMC were included in the data analysis. A combination of statistical and graphical normality tests showed continuous data for age, duration of service, duration of service in the psychiatric department, total HADS-A score, and total HADS-D score, and approach, avoidant and humour coping scores were normally distributed. In contrast, continuous data for religious coping score violated the normal distribution. 

Table 1 displays the socio-demographic, socio-economic, work-related factors, and coping strategies profiles of the respondents. The mean age of the respondents was 36.5 years old (SD = 8.1). The majority of the respondents were of Malay ethnicity (72.8%), female (68.2%), married (71.1%), and lived with their family members (82.7%). A total of 17.3% of respondents reported that their household income was negatively affected by the pandemic.

Forty-one per cent of the respondents were doctors, 36% were nurses, 18% were allied health professionals, and 5% were administrative staff. The allied health professionals were psychologists, counsellors, occupational therapists, physiotherapists, assistant medical officers, and healthcare assistants based at the Department of Psychological Medicine. More than half of the respondents reported an increased work burden during the pandemic (62.4%). More than 90% of the psychiatric HCWs reported having good social support (97.1%) and felt supported at their workplace (90.8%). Only around one-fourth (23.7%) of the respondents provided direct care to COVID-19 patients. More than four-fifths (88.4%) of the respondents had received their COVID-19 vaccination at the time of completion of the questionnaire. 

Twenty-two per cent (n = 38) of the respondents exhibited anxiety symptoms, while 16.7% (n = 29) of them reported depressive symptoms (Table 2). Eleven per cent (n = 19) of the respondents had both anxiety and depressive symptoms. Conversely, 72.3% (n = 125) of the psychiatric HCWs had neither anxiety nor depression.

Anxiety and depression were moderately and positively correlated with one another, with a correlation coefficient (r) value of 0.693 (*p* < 0.001). Anxiety was found to have a weak positive correlation with the avoidant coping domain (r = 0.394, *p* < 0.01) and a weak negative correlation with the religion coping domain (r = −0.177, *p* < 0.05). Depression only showed a significant positive correlation with the avoidant coping domain (r = 0.355, *p* < 0.01).

Table 3 illustrates the bivariate analysis between continuous and categorical variables associated with anxiety and depression. Female respondents, younger age, unmarried, staying alone, shorter duration of service, shorter duration of service in the psychiatric department, working longer hours, using a greater degree of avoidance coping, and a lesser degree of religious coping were significantly associated with more anxiety symptoms (*p*-value < 0.05). A shorter duration of service in the psychiatric department, staying alone, financial hardship, working longer hours, perceived as not being supported in the workplace, and a greater degree of avoidant coping were significantly associated with more depressive symptoms (*p*-value < 0.05). 

Table 4 shows the three significant predictors for anxiety and four significant predictors among the respondents in this study using multivariable logistic regression analysis, forward stepwise regression method. This model predicted 35% (Nagelkerke r^2^ = 0.352) and 30% (Nagelkerke r^2^ = 0.297) of the respondents as having anxiety and depressive symptoms, respectively. 

The married psychiatric HCWs had 3.3 times (*p* = 0.019) lower odds of having anxiety than the single respondents. The study also revealed that avoidant coping methods increase the respondent’s odds of having anxiety, whereas religious coping strategies reduce the respondent’s odds of having anxiety. For every increase of one unit of avoidant coping score among respondents, the odds of having anxiety increased by 1.25 times (*p* < 0.001), whereas for every increase of one unit of religious coping score among respondents, the odds of having anxiety decreased by 1.42 times (*p* = 0.008). 

The odds of depression among the respondents whose household financial situation worsened due to the pandemic were 3.7 times (*p* = 0.023) higher than those who were not financially affected during the COVID-19 pandemic. Aside from this, respondents who received the COVID-19 vaccination were 3.2 (*p* = 0.056) times less likely to be depressed than the unvaccinated respondents. With every increase in one unit of an avoidant coping score, the odds of becoming depressed increased by 20% (*p*-value < 0.001), whereas with every one month of service in the psychiatric department, the odds of psychiatric HCWs being depressed reduced by 1.2% (*p* = 0.003). 

## 4. Discussion

Contrary to the common misconception that psychiatric HCWs are resilient to the the adversity of the pandemic, this study found that the prevalence of anxiety and depressive symptoms among psychiatric HCWs was 22.0% and 16.7%, respectively. Psychiatric HCWs who were unmarried reported a greater degree of anxiety symptoms. Unvaccinated psychiatric HCWs, those experiencing financial hardships and those with a shorter duration of service in the psychiatric department had a higher level of depressive symptoms. The study also revealed that avoidant coping strategies predicted a higher level of anxiety and depressive symptoms, whereas religious coping strategies predicted lower levels of anxiety symptoms.

There were no local pre-pandemic studies in Malaysia evaluating the mental health of psychiatric HCWs for comparison. Nonetheless, pre-pandemic research in Greece by Papathanasiou et al. [41] showed that the level of anxiety and depression among psychiatric HCWs was 12.2% and 9.9%, respectively. The relatively higher prevalence of anxiety (22.0%) and depression (16.7%) in the present study highlights that psychiatric HCWs are not immune to the psychological effects of the pandemic.

The study’s prevalence of psychological distress is comparable to a multinational umbrella review of seven meta-analyses by Sahebi et al. [11]. Their review found that the prevalence of anxiety and depression among HCWs during the COVID-19 pandemic was 24.9% and 24.8%, respectively [11]. In a locally conducted study involving 200 frontline HCWs at the exact study location as the current study (UMMC) in April and May 2020, Chow et al. [42] revealed a much higher prevalence of anxiety and depression of 36.5% and 29.5%, respectively. However, this study was carried out within the first three months of the pandemic, whereas our study was conducted more than one year into the pandemic. There were likely different contributing stressors between the early and later phases of the COVID-19 pandemic. Similar changes in the prevalence of anxiety and depression over time among HCWs was also seen in other longitudinal studies during the COVID-19 pandemic [43,44]. 

The present study revealed that married psychiatric HCWs had a lower level of anxiety symptoms. Contradictory, Alnazly et al. [12] reported that married HCWs had significantly higher scores of depression, anxiety, and stress than single participants due to the fear of spreading the infection to their partners. Nonetheless, the same study also described those married participants as having better social support [12]. Similarly, several studies conducted during the COVID-19 pandemic documented that good social support was protective against developing anxiety [12,43]. The present study demonstrated that being married and having good social support has a greater protective effect more than the anxiety of infecting their spouse.

Another intriguing finding from the present study was that working in the psychiatric department was protective against developing depressive symptoms. This study revealed that for every added year of working in the psychiatric department, a person’s odds of developing depression reduced by around 15%. The findings coincided with a local study by Sahimi et al. [45] who, when evaluating the risk of suicidal ideation amongst HCWs serving during the pandemic, found that individuals with early career status (<10 years in service) were significantly at risk of having suicidal ideations compared to senior HCWs. The protective effect of having a longer service in the psychiatric department could be because of the lengthier work experience, higher salary, improved coping skills and better resilience. These findings suggest that it is crucial for the senior psychiatric HCWs, who are usually involved in service planning in the department, not to overlook the mental health state of their junior colleagues, despite most of the senior psychiatric HCWs not exhibiting any psychological distress.

The study also found that COVID-19 vaccination was a significant protective factor against depressive symptoms. The study period coincided with the HCWs at UMMC receiving their COVID-19 vaccine. A study conducted in Israel by Palgi et al. [19] revealed that high levels of vaccine hesitancy more than doubled the risk of depression (OR = 2.24) and more than tripled the risk for anxiety (OR = 3.62). The findings suggest that COVID- 19 vaccines, which have a high efficacy in preventing severe clinical disease and reducing the transmissibility of COVID-19, also reduce the psychological distress associated with COVID-19 infection among HCWs [46]. 

Although most psychiatric HCWs are employed by the government, and their salary remained unchanged during the pandemic, their spouse or family members’ income could have been negatively affected. The present study revealed that psychiatric HCWs with financial hardship caused by the COVID-19 pandemic had more than tripled odds of experiencing depression. This finding further supports the notion that economic wellbeing is vital for ensuring good mental health [47]. As per this finding, the financial implications of the pandemic on psychiatric HCWs should not be discredited simply because they are government employees. 

Regarding coping, similar to our study, local and international studies conducted during the COVID-19 pandemic also found that having a religious coping strategy was protective against developing anxiety [25,42,48,49]. To alleviate the negative consequences of chronic stress caused by the pandemic, people with positive religious coping strategies found it helpful to rely on a secure relationship with God/the divine and spiritual connectedness with others [25]. 

Conversely, the present study demonstrated that avoidant coping strategies were associated with an increased risk of developing anxiety and depression. This finding was in keeping with the association between coping strategies with anxiety and depression in a diverse sample of U.S. adults [50] and a local study among Malaysians during the COVID-19 pandemic [48]. Avoidance coping strategies may be beneficial for short-term uncontrollable stressors. Considering the COVID-19 pandemic is a chronic uncontrollable stressor, avoidance coping strategies would lead to more distress in the long term [23].

In keeping with data from other studies worldwide, most of the psychiatric HCWs in our study also reported an increase in work burden [7,51]. However, our study did not show any statistically significant association between increasing work burden with anxiety or depression. The good support at work reported by 90% of the respondents likely mitigated the psychological distress due to the increased work burden. A supportive workplace environment reduces occupational stress and is crucial for self-efficacy and professional identity [18,52].

The analysis also did not find any association between providing direct care for COVID-19 patients and having psychological distress. This could be because the hospital provides intensive training on infection control measures and adequate support for all HCWs caring for COVID-19 patients. HCWs who have acquired the necessary knowledge, skills, and training to manage COVID-19 patients with a lower risk of psychological distress [53]. Similar to the present study, Norhayati et al. [54] conducted a local study in Kelantan, comparing depressive symptoms among frontline and non-frontline HCWs. Norhayati et al. [54] found that non-frontline HCWs exhibited higher depressive symptoms (37.7%) than frontline healthcare providers (27.5%). These findings further highlight the importance of not neglecting the mental health of HCWs who are not directly involved with caring for COVID-19 patients. 

The significant correlation between coping strategies, vaccination, marital status, financial difficulties, and duration of service in psychological wellbeing in the present study sheds new light on providing more intensive, targeted, psychological interventions for psychiatric HCWs at high risk of developing psychological distress. From observing the relatively high prevalence of anxiety and depression among psychiatric HCWs, a combined approach consisting of organizational interventions and targeted individual psychological support should be in place to alleviate the psychological impact of the pandemic among all psychiatric HCWs [55]. If psychological distress among psychiatric HCWs is left unaddressed, their work productivity may be impaired, ultimately resulting in suboptimal patient care. Early intervention could help avert mental health complications among psychiatric HCWs, while preserving essential mental health services during the pandemic.

There are a few limitations to this study. The study’s cross-sectional design could only identify associations between variables and renders the study of causality implausible. Additionally, as the study was limited to the psychiatric HCWs in a single teaching hospital, the prevalence or findings of the study population might not represent all the psychiatric HCWs in Malaysia. Furthermore, conducting the study during a short time frame, from 1 May 2021 to 31 August 2021, also renders the prevalence of the study population less accurate for other periods of the pandemic, considering the prolonged duration and differing challenges in different periods of the COVID-19 pandemic. 

A cohort or qualitative experimental study design would be beneficial to further evaluate the relationships between the variables in this study and anxiety and depression. It would have been ideal if the study incorporated psychiatric HCWs across multiple government and private hospitals and surveyed them at different periods of the pandemic. The study also did not include burnout, which could be a confounding factor to anxiety and depression [10,17,18]. Burnout among psychiatric HCWs could further enlighten the association of work-related factors with anxiety and depression. Nonetheless, due to time limitations and the standard operating practices requirement for studies during the pandemic, this study was safer and had the strength to explore associations between various factors. 

## 5. Conclusions

To the authors’ knowledge, this is the first study evaluating the psychological impact of the COVID-19 pandemic among the psychiatric HCWs in Malaysia. While much emphasis has been devoted to the mental health of frontline HCWs, the present study establishes that the COVID-19 pandemic impacted the psychological wellbeing of (non-frontline) psychiatric HCWs. The study found a relatively high level of anxiety and depression among psychiatric HCWs, comparable to the psychological distress experienced by frontline HCWs. Thus, psychosocial measures addressing the mental health of psychiatric HCWs should be a key priority as they play a vital role in the care of other HCWs and patients with mental health difficulties in the face of this prolonged pandemic.

## Figures and Tables

**Table 1:** Socio-demographic, socio-economic, work-related factors, and coping strategies profiles among psychiatric healthcare workers at University Malaya Medical Centre (n = 173).

Variables		n (%)
**Age (years) ^a^**		36.46 (8.05)
**Duration of service (months) ^a^**		147.53 (90.36)
**Duration of service in psychiatric department (months) ^a^**		103.57 (86.55)
**Ethnicity**		
Malay		126 (72.8)
Non-Malay		47 (27.2)
**Gender**		
Female		118 (68.2)
Male		55 (31.8)
**Education attainment**		
Degree and higher		87 (50.3%)
Diploma and lower		86 (49.7%)
**Medical comorbidity**		
No		148 (85.5)
Yes		25 (14.5)
**Psychiatric history**		
No		126 (72.8)
Yes		47 (27.2)
**Marital status**		
Single		44 (25.4)
Married		123 (71.1)
Divorce		6 (3.5
**Number of children**		
At least one child		108 (62.4)
No children		65 (37.6)
**Living arrangements**		
Alone		13 (7.5)
Family		143 (82.7)
Friend(s)		17 (9.8)
**Living with elderly**		
No		131 (75.7)
Yes		42 (24.3)
**Family member tested positive for COVID-19**		
No		138 (79.8)
Yes		35 (20.2)
**Household income**		
(n = 171)		
Less than RM 4850 (B40 income tier)		43 (25.1)
RM 4850–RM 10,959 (M40 income tier)		98 (57.3)
More than RM 10,959 (T20 income tier)		30 (17.5)
**Financial hardship during COVID-19**		
No		126 (72.8)
Yes, getting worse		47 (27.2)
**Adequate social support**		
No		5 (2.9)
Yes		168 (97.1)
**Occupation**		
Doctor		71 (41.0)
Nurse		62 (35.8)
Allied Health Professional		31 (17.9)
Administrative Staff		9 (5.2)
**Work in shift rotation**		
No		106 (61.3)
Yes		67 (38.7)
**Working hours per week**		
45 h or less		123 (71.1)
46 h or more		50 (28.9)
**Providing direct care to COVID-19 patients**		
No		132 (76.3)
Yes		41 (23.7)
**Adequate support at workplace**		
No		16 (9.2)
Yes		157 (90.8)
**Increased work burden due to COVID-19 pandemic**		
No		65 (37.6)
Yes		108 (62.4)
**Investigated as close contact for COVID-19**		
No		117 (67.6)
Yes		56 (32.4)
**Tested for COVID-19**		
No		106 (61.3)
Yes		67 (38.7)
**Tested positive with COVID-19**		
No		169 (97.7)
Yes		4 (2.3)
**Perceived at risk group for COVID-19**		
No		31 (17.9)
Yes		142 (82.1)
**Received COVID-19 vaccination**		
No		20 (11.6)
Yes		153 (88.4)
**Coping**	Approach ^a^	33.63 (9.03)
	Avoidant ^a^	21.44 (5.48)
	Religion ^b^	7 (3)
	Humour ^a^	4.57 (1.70)

^a^: Mean (standard deviation, SD). ^b^: Median (interquartile range, IQR).

**Table 2:** Prevalence of anxiety and depression symptoms among psychiatric healthcare workers at University Malaya Medical Centre (n = 173).

Scale	Prevalence (n)	Lower 95% CI	Higher 95% CI
**HADS-Anxiety**			
Minimal/No (<8)	78.0% (135)	71.3%	83.6%
Mild (8–10)	16.8% (29)	11.9%	23.0%
Moderate (11–14)	4.0% (7)	2.0%	8.1%
Severe (15–21)	1.2% (2)	0.3%	4.1%
**HADS-Depression**			
Minimal/No (<8)	83.2% (144)	77.0%	88.1%
Mild (8–10)	12.7% (22)	8.6%	18.5%
Moderate (11–14)	4.0% (7)	2.0%	8.1%
Severe (15–21)	0.0% (0)		
**HADS-Anxiety and Depression**			
Neither anxiety nor depression	72.3% (125)	65.2%	78.4%
Either anxiety or depression	16.8% (29)	11.9%	23.0%
Both anxiety and depression	11.0% (19)	7.1%	16.5%

CI: Confidence interval.

**Table 3:** Bivariate analysis for socio-demographic, socio-economic, work-related factors, and coping strategies associated with anxiety and depression among psychiatric healthcare workers at University Malaya Medical Centre (n = 173).

Variables		Bivariate Analysis					
		Anxiety			Depression		
		HADS-A (Score > 8) n (%)	HADS-A (Score < 8) n (%)	*p*-Value	HADS-D (Score > 8) n (%)	HADS-D (Score < 8) n (%)	*p*-Value
**Age (years) ^a^**		34.3 (7.1)	37.0 (8.2)	0.047 *	34.3 (6.2)	36.9 (8.3)	0.057 *
**Duration of service (months) ^a^**		120.6 (86.1)	155.1 (90.4)	0.037 *	119.1 (74.8)	153.3 (92.3)	0.063 *
**Duration of service in psychiatric department (months) ^a^**		64.4 (69.0)	114.6 (88.0)	<0.001 *	60.8 (59.1)	112.2 (88.8)	<0.001 *
**Ethnicity ^c^**				0.337			0.188 *
Malay		30 (78.9)	96 (71.1)		24 (82.8)	102 (70.8)	
Non-Malay		8 (21.1)	39 (28.9)		5 (17.2)	42 (29.2)	
**Gender ^c^**				0.020 *			0.733
Female		20 (52.6)	98 (72.6)		19 (65.5)	99 (68.8)	
Male		18 (47.4)	37 (27.4)		10 (34.5)	45 (31.3)	
**Education attainment ^c^**				0.153 *			0.519
Degree and higher		23 (60.5)	64 (47.4)		13 (44.8)	74 (51.4)	
Diploma and lower		15 (39.5)	71 (52.6)		16 (55.2)	70 (48.6)	
**Medical comorbidity ^c^**				0.193 *			0.259
No		35 (92.1)	113 (83.7)		27 (93.1)	121 (84.0)	
Yes		3 (7.9)	22 (16.3)		2 (6.9)	23 (16.0)	
**Psychiatric history ^d^**				0.122 *			0.425
No		36 (94.7)	134 (99.3)		28 (96.6)	142 (98.6)	
Yes		2 (5.3)	1 (0.7)		1 (3.4)	2 (1.4)	
**Marital status c**				0.004 *			1.000
Single (Ref)		16 (42.1)	28 (20.7)		7 (24.1)	37 (25.7)	
Married		19 (50.0)	104 (77.0)		21 (72.4)	102 (70.8)	
Divorce		3 (7.9)	3 (2.2)		1 (3.4)	5 (3.5)	
**Number of children ^c^**				0.073 *			0.707
At least one child		19 (50.0)	89 (65.9)		19 (65.5)	89 (61.8)	
No children		19 (50.0)	46 (34.1)		10 (34.5)	55 (38.2)	
**Living arrangements ^c^**				0.023 *			0.023 *
Alone		6 (15.8)	7 (5.2)		5 (17.2)	8 (5.6)	
Family		26 (68.4)	117 (86.7)		24 (82.8)	119 (82.6)	
Friend(s)		6 (15.8)	11 (8.1)		0 (0)	17 (11.8)	
**Living with elderly ^c^**				0.600			0.649
No		30 (78.9)	101 (74.8)		21 (72.4)	110 (76.4)	
Yes		8 (21.1)	34 (25.2)		8 (27.6)	34 (23.6)	
**Family member tested positive for COVID-19 ^c^**				0.549			0.660
**No**		29 (76.3)	109 (80.7)		24 (82.8)	114 (79.2)	
**Yes**		9 (23.7)	26 (19.3)		5 (17.2)	30 (20.8)	
**Household income c (n = 171)**				0.175 *			<0.022 *
Less than RM 4850		12 (31.6)	31 (23.3)		8 (27.6)	35 (24.6)	
RM 4850–RM 10,959		23 (60.5)	75 (56.4)		21 (72.4)	77 (54.2)	
More than RM 10,959		3 (7.9)	27 (20.3)		0 (0.0)	30 (21.1)	
**Financial hardship ^c^**				0.209 *			0.110 *
No		34 (89.5)	109 (80.7)		21 (72.4)	122 (84.7)	
Yes, getting worst		4 (10.5)	26 (19.3)		8 (27.6)	22 (15.3)	
**Adequate social support ^d^**				0.303			0.591
No		2 (5.3)	3 (2.2)		0 (0.0)	5 (3.5)	
Yes		36 (94.7)	132 (97.8)		29 (100.0)	139 (96.5)	
**Occupation ^c^**				0.052 *			0.919
Doctor		22 (57.9)	49 (36.3)		13 (44.8)	58 (40.3)	
Nurse		8 (21.1)	54 (40.0)		9 (31.0)	53 (36.8)	
Allied Health Professional		5 (13.2)	26 (19.3)		5 (17.2)	26 (18.1)	
Administrative Staff		3 (7.9)	6 (4.4)		2 (6.9)	7 (4.9)	
**Work in shift rotation ^c^**				0.306			0.460
No		26 (68.4)	80 (59.3)		16 (55.2)	90 (62.5)	
Yes		12 (31.6)	55 (40.7)		13 (44.8)	54 (37.5)	
**Working hours per week ^c^**				0.042 *			0.038 *
45 h or less		22 (57.9)	101 (74.8)		16 (55.2)	107 (74.3)	
46 h or more		16 (42.1)	34 (25.2)		13 (44.8)	37 (25.7)	
**Providing direct care to COVID-19 patients ^c^**				0.998			0.590
No		29 (76.3)	103 (76.3)		21 (72.4)	111 (77.1)	
Yes		9 (23.7)	32 (23.7)		8 (27.6)	33 (22.9)	
**Adequate support at workplace ^d^**				0.303			0.031 *
No		7 (18.4)	9 (6.7)		6 (20.7)	10 (6.9)	
Yes		31 (81.6)	126 (93.3)		23 (79.3)	134 (93.1)	
**Increased work burden due to COVID-19 pandemic ^c^**				0.214 *			0.224 *
No		11 (28.9)	54 (40.0)		8 (27.6)	57 (39.6)	
Yes		27 (71.1)	81 (60.0)		21 (72.4)	87 (60.4)	
**Investigated as close contact for COVID-19 ^c^**				0.147 *			0.116 *
No		22 (57.9)	95 (70.4)		16 (55.2)	101 (70.1)	
Yes		16 (42.1)	40 (29.6)		13 (44.8)	43 (29.9)	
**Tested for COVID-19**				0.629			0.923
**No**		22 (57.9)	84 (62.2)		18 (62.1)	88 (61.1)	
**Yes**		16 (42.1)	51 (37.8)		11 (37.9)	56 (38.9)	
**Tested positive with COVID-19 ^d^**				0.210 *			0.523
No		36 (94.7)	133 (98.5)		28 (96.6)	141 (97.9)	
Yes		2 (5.3)	2 (1.5)		1 (3.4)	3 (2.1)	
**Perceived at risk group for COVID ^c^**				0.569			0.917
No		8 (21.1)	23 (17.0)		5 (17.2)	26 (18.1)	
Yes		30 (78.9)	112 (83.0)		24 (82.8)	118 (82.0)	
**Received COVID-19 vaccination ^d^**				0.391			0.111 *
No		6 (15.8)	14 (10.4)		6 (20.7)	14 (9.7)	
Yes		32 (84.2)	121 (89.6)		23 (79.3)	130 (90.3)	
**Coping**	Approach ^a^	34.1 (6.8)	33.5 (9.6)	0.661	33.3 (7.9)	33.7 (9.3)	0.818
	Avoidant ^a^	24.8 (5.2)	20.5 (5.2)	<0.001 *	25.0 (5.7)	20.7 (5.1)	<0.001 *
	Religion ^b^	6 (3.0)	7 (2.0)	0.002 *	6 (3.0)	7 (3.0)	0.812
	Humour ^a^	4.5 (1.5)	4.6 (1.7)	0.767	4.4 (1.6)	4.6 (1.7)	0.583

^a^: Mean (SD), independent *t*-test. ^b^: Median (IQR) Mann–Whitney U test. ^c^: Pearson chi-square test. ^d^: Fisher’s exact test. * *p* < 0.25 (selected for multivariate analysis).

**Table 4:** Multiple logistic regression analysis of factors associated with anxiety and depression among psychiatric healthcare workers at University Malaya Medical Centre (n = 173).

Variables	Anxiety		Depression	
	Adjusted B (95% CI)	*p*-Value	Adjusted B (95% CI)	*p*-Value
**Marital status**			-	-
Married		0.014 *		
Divorce	0.306 (0.114, 0.826)	0.019 *		
Single (Ref)	3.030 (0.448, 20.499)	0.256		
**(Coping) Religion**	0.704 (0.543, 0.913)	0.008	-	-
**(Coping) Avoidant**	1.254 (1.145, 1.373)	<0.001 **	1.199 (1.093, 1.315)	<0.001 **
**Financial hardship**	-	-	3.666 (1.193, 11.268)	0.023 *
Yes				
No (Ref)				
**Received COVID-19 vaccination**	-	-	0.312 (0.095, 1.03)	0.056
Yes				
No (Ref)				
**Duration of service in psychiatric department (months)**	-	-	0.988 (0.981, 0.996)	0.003 **

B: Regression coefficient. CI: Confidence interval. (Ref): Reference group. * *p* < 0.05. ** *p* < 0.001.

## Data Availability

The data presented in this study are available on request from the corresponding author. The data are not publicly available due to privacy and ethical issues.
